# Acute mountain sickness among tourists visiting the high-altitude city of Lhasa at 3658 m above sea level: a cross-sectional study

**DOI:** 10.1186/s13690-016-0134-z

**Published:** 2016-06-01

**Authors:** Gonggalanzi, Labasangzhu, Per Nafstad, Hein Stigum, Tianyi Wu, Øyvind Drejer Haldorsen, Kristoffer Ommundsen, Espen Bjertness

**Affiliations:** Institute of Health and Society, University of Oslo, P.O. Box 1130 Blindern, Oslo, 0318 Norway; Tibet University Medical College, No. 1 South Luobulinka Road, Lhasa, 850002 Tibet China; Division of Epidemiology, Norwegian Institute of Public Health, Oslo, Norway; National Key Laboratory of High-Altitude Medicine, Qinghai, China

**Keywords:** Acute mountain sickness, Tourist, Tibet

## Abstract

**Background:**

Traveling to Tibet implies a risk for developing acute mountain sickness (AMS), and the size of this problem is likely increasing due to the rising number of tourists. No previous study on AMS has been conducted among the general tourist population in Tibet. Thus, the aim of this study was to estimate the prevalence and determinants of AMS in a large tourist population visiting Lhasa.

**Methods:**

A sample of 2385 tourists was recruited from seven randomly selected hotels in Lhasa between June and October 2010. Within three days of their first arrival, the participants filled in a questionnaire based on the Lake Louise Scoring System (LLSS) about AMS-related symptoms and potential contributing factors. AMS was defined as the presence of headache and a cumulative Lake Louise Score ≥4. After estimating the prevalence of AMS, a Log-Binomial Model was applied to analyse the relationship between AMS and selected risk factors.

**Results:**

The prevalence of AMS was 36.7 % (95 % CI: 34.6–38.7 %) and was not dependent on tourists’ country of origin. Among the participants who developed AMS, 47.6 % reported that they experienced symptoms within the first 12 h after arriving in Lhasa, and 79.0 % reported that they had to reduce their activity level. A poor or average health condition (adjusted PR 1.63, 95 % CI 1.38–1.93), an age below 55 years (adjusted PR 1.29, 95 % CI 1.04–1.60), a rapid ascent to Lhasa (adjusted PR 1.17, 95 % CI 1.02–1.34) were independent AMS risk factors, while smoking (adjusted PR 0.75, 95 % CI 0.59–0.96) and pre-exposure to high altitude (adjusted PR 0.71, 95 % CI 0.60–0.84) reduced the risk of AMS.

**Conclusions:**

AMS is commonly experienced by tourists visiting Lhasa Tibet, and often affects their activities. The tourists’ country of origin did not seem to affect their risk of AMS, and their age was inversely related to AMS. Subjects planning to visit a high-altitude area should be prepared for experiencing AMS-related problems, and consider preventive measures such as pre-exposure or a gradual ascent to high altitudes.

## Background

Since the opening of the world’s highest plateau railway (The Qinghai-Tibet Railway) to Lhasa in 2006, the number of tourists visiting Tibet has increased sharply from 2.5 million in 2006 to more than 15 million visitors in 2014 [1]. Tourism revenue accounted for over 20 % of the region’s gross domestic product (GDP) in 2014 [1]. Tourists travelling to Tibet from low-altitude areas have the potential risk of developing acute mountain sickness (AMS) during the first few days due to exposure to hypobaric hypoxia environment at high altitude [2]. AMS is usually characterized by symptoms of headache, dizziness, vomiting, anorexia, fatigue and insomnia after arrival at high altitudes [3]. In some serious cases, AMS can progress to life-threatening high-altitude cerebral edema (HACE) or high-altitude pulmonary edema (HAPE) [4]. The prevalence of AMS after ascending to high altitude has been reported to vary between 9 and 84 % [5–9]. There are few places where large numbers of ordinary tourists can easily and rapidly reach altitudes as high as in Lhasa (3658 m above sea level). A recent study reported that 51 % of construction workers experienced AMS upon first-time exposure to high altitude on the Qinghai-Tibet railroad route [10]. In addition, 57 % of army recruits who travel from the lowlands to Lhasa by air developed AMS [11]. These studies were carried out in quite homogenous populations, primarily consisting of young participants in presumably good health, which is probably different from what one would expect to be the case among ordinary tourists visiting Lhasa nowadays. Data on AMS among ordinary tourists is scarce. More knowledge about AMS among tourists travelling to high altitude could be important for persons planning to go there, as well as for professionals taking care of them both before and after arrival at such altitudes. The present study aimed to estimate the prevalence of AMS and to identify the determinants for developing AMS in an adult population of ordinary tourists visiting Lhasa Tibet China.

## Methods

### Ethics

The Ministry of Health and the Tibet University Medical College in TAR approved the research. The study protocol was submitted to the Norwegian National Committee for Medical and Health Research Ethics, which found the study unnecessary to undergo evaluation since the collected information was considered anonymous. Information about the details of the study was given on the first page of the questionnaire. The potential participants were also informed that the study was voluntarily and anonymous, and that they could refuse to participate without any negative consequences.

### Setting

The data collection was carried out in Lhasa, the capital city of the Tibet Autonomous Region (TAR). The elevation of Lhasa makes it one of the highest situated cities in the world [1]. Lhasa is a sacred city situated in the Himalayas and attractive for many types of tourists. There were 90 star-rated hotels and a few guest houses in Lhasa at the time of the data collection [12].

### Study samples

Nine hotels were randomly selected to participate in the study from a list covering all hotels in Lhasa which was given by the local tourism bureau. The management of the hotels were informed about the study and asked if they were willing to participate. All hotels agreed to participate, but the managers of two of the hotels decided to withdraw from the study when the data collection started. Thus, in seven hotels, tourists older than 15 years of age were invited to participate in the present study between 2 June and 31 October 2010. Receptionists from the selected hotels were given instructions in how to inform participants about the study, all aspects of the data collection as well as how to act in case study participants were in need of support for AMS related problems. Tourists received a questionnaire and instructions about the criteria needed for participation (age >15 years; classified as a tourist), and how to fill in information and return the questionnaire before leaving the hotel, depending on the duration of their stay at the hotel. This meant that the questionnaire was to be returned to the receptionist the third night after their arrival, or on the day when they checked out if the tourists planned to stay two nights or less. In total, 2385 tourists were invited to participate during the data collection period and all returned the questionnaire. It turned out that 106 of the tourists refused to participate, and handed in their questionnaire without information. The lack of an understanding Chinese and English and limited time were the main reasons for refusing to participate. Furthermore, 76 participants were excluded due to incomplete questionnaire information, non-tourist status or an age below 15 years, leaving 2203 participants (92.4 %) for the analyses.

### Variables

The questionnaire was tested in a pilot study in 2008, and a revised version was available for the current study in both Chinese and English. The questionnaire was designed to obtain data concerning age, gender, height, weight, altitude of permanent residence, nationality, education, type of transport to Lhasa (by plane, by train, by bus or by car), previous exposure to high altitude, prior history of high-altitude illness, the use of prophylactic medicine, smoking habits, awareness of altitude sickness and self-reported health condition. Body Mass Index (BMI) was calculated as body weight (kg) divided by height (m) squared. According to the World Health Organization (WHO), obesity was categorized by BMI ≥ 30.00.

AMS was assessed by the Lake Louise Score System (LLSS) [13], which is based on the most frequent symptoms considered important for AMS: headache, dizziness, gastrointestinal distress (loss of appetite, nausea, or vomiting), lassitude or fatigue and insomnia [14]. Each item is scored by the subject on a scale between 0 and 3 (0 = none, 1 = mild, 2 = moderate, 3 = severe). Single item scores are added up, with the total scores ranging from a minimum of 0 to a maximum of 15. Headaches have been recognized as a key symptom of AMS by previous researchers [15–17]. AMS was defined as the presence of a headache, at least one other symptom and a total LLS ≥4 [14].

### Statistical analysis

The Log-Binomial Model was applied to analyse the relationship between AMS and selected risk factors. The crude and adjusted prevalence ratio (PR) was computed with 95 % confidence intervals (Cl), level of statistical significance was set at *p* < 0.05. The analyses were carried out using SPSS (Statistical Package for Social Sciences, Version 22 for Windows. SPSS Inc. Chicago, USA, 2010).

## Results

### Population characterization

The participants originated from 48 different countries. The largest group was from China (46.9 %), followed by the Netherlands (6.3 %), the US (5.8 %), Germany (4.8 %), France (3.7 %) and the United Kingdom (3.7 %). Population characteristics are given in Table 1, and there was an approximately equal representation of men and women, and the mean age was 37.2 ± 14.4 years (range 15–81 years). Most of the tourists were non-obese, lived at low altitudes, non-smokers and considered themselves to be in good health. Almost half of the participants took prophylactic medicine. Among them, 72.6 % took Rhodiola or other Chinese medicine, 25.3 % used acetazolamide or diamox and 2.1 % used steroids or nifedipin. More than one-third of the participants reported previous AMS symptoms, and 25.6 % had been exposed to high altitudes in the preceding three months. Some tourists reported to have diabetes mellitus (*n* = 30;1.4 %), high blood pressure (*n* = 112;5.3 %), cardio-vascular disease (*n* = 25;1.2 %) or lung diseases (*n* = 90;4.3 %).

**Table 1:** Characteristics, prevalence and risk factors of acute mountain sickness among tourists above 15 years of age arriving in Lhasa, Tibet, China between June and October 2010

Characteristics	N	AMS+	Crude PR	Adjusted PR*
		N (%)	(95 % CI)	(95 % CI)
Gender				
Female	1072	387 (36.1)	1	1
Male	1103	461 (41.8)	1.16 (1.04,1.29)	1.08 (0.94,1.23)
Age				
≥ 55 years	360	116 (32.2)	1	1
< 55 years	1796	722 (40.2)	1.25 (1.06,1.46)	1.29 (1.04,1.60)
Obesity				
No	2015	787 (39.1)	1	1
Yes	119	48 (40.3)	1.03 (0.82,1.29)	1.02 (0.76,1.37)
Nationality				
Chinese	1018	401 (39.4)	1	1
Other nationalities	1162	447 (38.5)	0.98 (0.88,1.09)	0.92 (0.78,1.08)
Altitude of permanent residence/home				
2000 m or higher (>6500 ft)	111	37 (33.3)	1	1
Below 2000 m (<6500 ft)	1994	784 (39.3)	1.18 (0.90,1.54)	1.06 (0.76,1.49)
Education				
College or higher	1766	701 (39.7)	1	1
High school or lower	361	130 (36.0)	1.10 (0.95,1.28)	1.11 (0.92,1.33)
Smoking				
No	1849	742 (40.1)	1	1
Yes	251	83 (33.1)	0.76 (0.63,0.92)	0.75 (0.59,0.96)
Transportation				
Not by air	1138	409 (35.9)	1	1
By air	1022	435 (42.6)	1.18 (1.07,1.32)	1.17 (1.02,1.34)
Health condition				
Good health	1904	710 (37.3)	1	1
Poor or average health	197	117 (59.4)	1.59 (1.40,1.81)	1.63 (1.38,1.93)
Previous AMS symptoms				
No	1011	361 (35.7)	1	1
Yes	590	221 (37.5)	1.05 (0.92,1.20)	1.10 (0.96,1.26)
Awareness of AMS				
No	248	92 (37.1)	1	1
Yes	1851	732 (39.5)	0.94 (0.79,1.11)	1.02 (0.83,1.27)
Pre-exposure in the preceding 3 months				
No	1609	695 (43.2)	1	1
Yes	552	148 (26.8)	0.62 (0.54,0.72)	0.71 (0.60,0.84)
Use of prophylactic				
No	1116	416 (37.3)	1	1
Yes	965	399 (41.3)	1.11 (1.00,1.24)	1.05 (0.92,1.20)

*Adjusted for all variables in the table

### Acute mountain sickness

A total of 808 (36.7 %, CI: 34.6–38.7 %) subjects reached the standard of AMS with headache and a total LLS ≥ 4. Table 1 also shows the prevalence of AMS in different subgroups of the population, as well as crude and adjusted prevalence ratios for the potential determinants of AMS. Different approaches of analyses yielded similar results: analyses based on including all determinants in the model, specific causal models for each of the variables and models that only included variables that were statistically significantly related to AMS in the crude analyses. Only results from the first approach are reported in the present study, and we did not find any substantial differences in AMS prevalence between Chinese and other nationalities. Further stratification into participants from Asia, Europe, America and Oceania did not show a substantial variation in AMS prevalence either. Moreover, we did not find contrasts in prevalences of AMS between users and none users of prophylactic medicine. That was also the case for subgroups of participants who used Rhodiola, acetazolamide, steroids and nifedipin (results not given). In the fully adjusted model, the factors that remained statistically significantly associated with a higher risk of AMS were a poor or average health condition, no pre-exposure to high altitude, an age below 55 years, being a non-smoker and arrival in Lhasa by air.

**Fig. 1:**
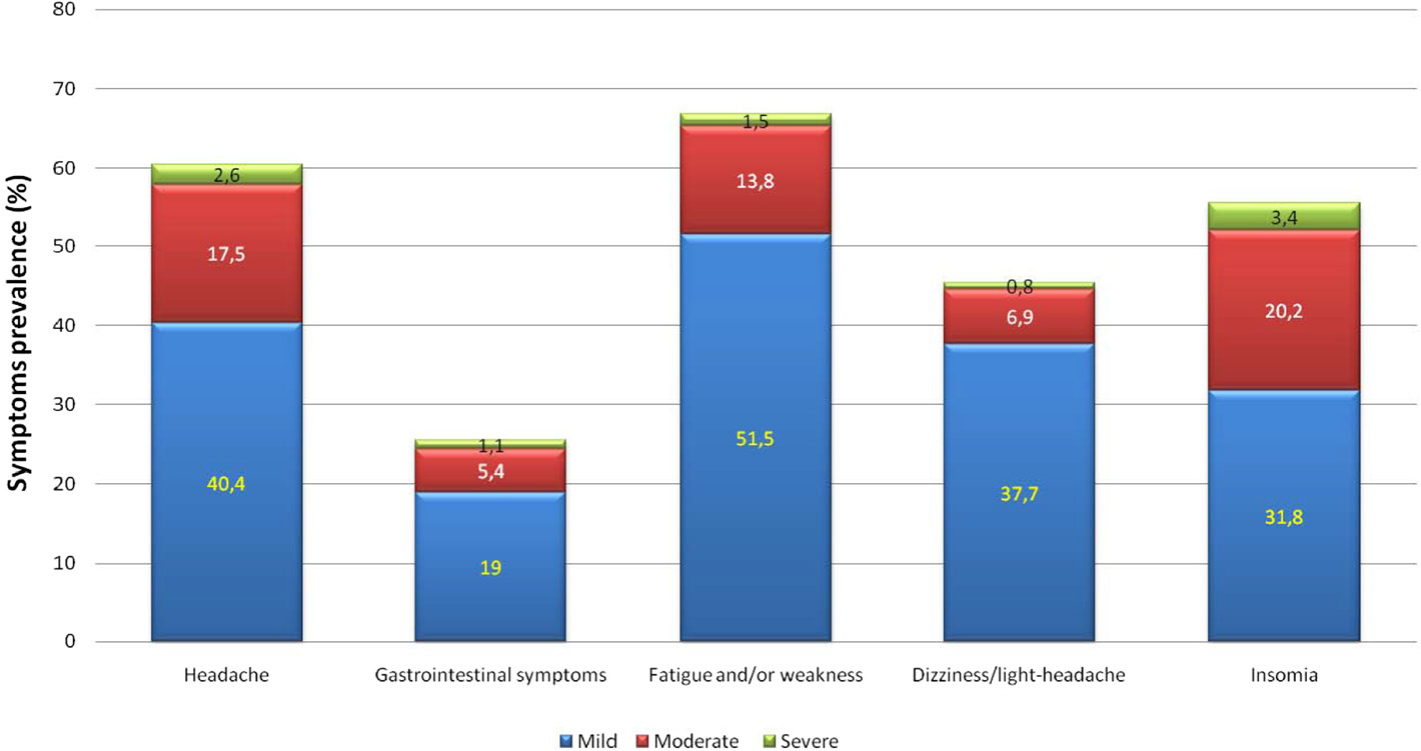
Prevalence of symptoms of acute mountain sickness by severity among tourists above 15 years of age arriving in Lhasa, Tibet, China between June and October 2010

A total of 1808 participants (82.1 %) reported at least one of the recorded symptoms. Fatigue was the most frequently reported symptom, followed by headache, insomnia, dizziness and gastrointestinal symptoms (Fig. 1). The mean overall AMS scores were 3.34 ± 2.63. In subgroups among those both with and without AMS, the mean scores differed significantly (*p* < 0.05), 5.94 ± 1.92 and 1.68 ± 1.40, respectively. In 30.5 % of participants, the onset of AMS symptoms started as early as on the journey to Lhasa, while in 47.6 % of participants the symptoms started within the first 12 h after arrival and in 21.9 % after 12 h. Furthermore, 21.0 % of the participants with AMS did not reduce their physical activity and 74.0 % had a moderate activity reduction, while 5.0 % chose to rest in bed because of symptoms. A total of 282 participants reported to have sought help or advice for AMS. Among them, 150 (53.2 %) got help from their tour companions or local friends, 92 (32.6 %) went to local hospitals and 20 (7.1 %) received a visit by the local doctor. No cases of HACE or HAPE were reported among the participants during the three days follow up.

## Discussion

AMS defined as LLS ≥ 4 with headache was reported by 36.7 % of the participants. Fatigue, headache and insomnia were the three most commonly reported AMS related symptoms (66.8, 60.4 and 56.3 %, respectively). Tourists who reported to not be in a good health condition, to have no pre-exposure at high altitude in the preceding three months, to be younger than 55 years of age, to be a non-smoker and to have ascended to high altitude by air were at increased risk of experiencing AMS.

To the best of our knowledge, no other study has addressed the prevalence and risk factors of AMS in a large population of ordinary tourists arriving at an altitude as high as that in Lhasa. Characteristics of tourists visiting Lhasa will most likely change over time. We believe that the current study population resembled the typical recreational tourists to Tibet during the data collection period. The population showed a broad variation in characteristics that might influence their risk of developing AMS.

AMS was definitely a common problem among the study participants. Previous studies have reported both a higher and lower prevalence of AMS than our finding [6, 18–21]. Comparisons between studies are complicated by differences in population characteristics [20], altitude reached [6] and AMS definitions [18]. The current AMS prevalence is lower than what was reported in two other studies from Tibet, including one among tourists (42.3 %) visiting the Namtso Lake at 4718 m in Tibet [22] and one among construction workers (51 %) at Qinghai-Tibet altitudes up to 5000 m [23]. The higher altitude in these two studies could explain the higher prevalence of AMS compared to our study. The prevalence in our study was clearly higher than in a study carried out among tourists (28 %) in La Paz Bolivia at a similar altitude (3630 m) as that of Lhasa, even if we used a stricter definition of AMS [24]. However, that study consisted of only 32 participants, and AMS was measured immediately after arrival at high altitude. Several studies have been conducted among trekkers and mountaineers [8, 18, 20, 25, 26] with varying results. For example, one study showed a prevalence of 34 % in mountaineers in the Alps [8], which is quite comparable to our findings, while another study reported a prevalence of 10 % in trekkers in the Nepali Himalaya [18]. Overall, we believe that the prevalence found in this study is of an expected size compared with earlier studies if we attempt to take into account the differences in study populations, the altitude reached and the disease definition.

The unique culture and sacred places in Lhasa may motivate a variety of tourists to go there. Based on our observations, we cannot conclude that a population consisting of ordinary tourists has a substantially higher risk of AMS than more selected groups, even if there is reason to believe that such a population is less healthy and less physically fit compared to trekkers [11], mountaineers [25] and construction workers [10]. Our findings support the idea that AMS symptoms typically appear within the first 12 h after arrival at high altitude [3, 27, 28], and that people from different countries have a similar risk of developing AMS. In the present study, fatigue was the most frequently reported symptom of AMS, followed by headaches and sleep disorders. This is somewhat inconsistent with some earlier studies [16, 28, 29] that reported headaches and sleep disorders to be more common than fatigue. However, all these symptoms were common and most tourists have to expect some AMS-related symptoms and a reduction of activity during the first days, in addition to a few who would also prefer to stay in bed.

A lack of pre-exposure and rapid ascent to high altitude have been linked to AMS in previous studies [9, 11, 30–32]. For instance, Schneider and colleagues [32] reported that altitude exposure in the preceding two months reduces the risk of AMS. Hultgren and colleagues [33] found climbers who visited the Himalayas annually had fewer symptoms and improved their physical performance compared with the first time they visited. Our findings corroborate with this, and indicate some degree of physiological acclimatization and residual benefit from high-altitude exposure during the last three months. Living at high altitude has also been found to protect against AMS [6, 34]. Only a small proportion of the participants in our study lived at an altitude above 2000 m. Even if the AMS prevalence was low within this group, a low statistical power made it difficult to draw firm conclusions based on our findings. Several studies have shown an increased risk of AMS with a rapid ascent to high altitude [2, 3, 9]. Murdoch and colleagues found the prevalence of AMS to be 84 % among tourists who flew directly to Shyangboche at the altitude of 3740 m compared with 61 % among those who walked up from altitudes under 3000 m to the same altitude [9]. Our findings support this idea, as persons arriving by plane reported more frequent AMS than others. It is a possibility that the chosen transport to high altitude is related to a person’s risk of developing AMS. We have attempted to address this by adjusting for an awareness of AMS and previous experience of AMS in the analyses. A further selection effect would probably lead to an underestimation of the effect of rapid ascent.

The impact of health conditions on the development of AMS is of importance for individual’s decisions to travel to high altitude, though the answer to this question is unclear [6, 14, 31]. The prevalence of AMS was similar between those with and without diseases such as lung disease and cardio-vascular disease. This observation is in accordance with previous studies (18, 28, 29). On the other hand, we found that those who reported not being in good health had a higher risk of AMS. This seeming inconsistency could be explained by subjective reports of health condition by participants. Although smoking is generally considered harmful to health, studies have not always confirmed this [35, 36]. For example, Wu and colleagues [35] found that an 11 % decrease in the prevalence of AMS in smokers compared with non-smokers, whereas Song and colleagues [36] found that the prevalence of AMS was lower in smokers than in non-smokers. Our finding is in agreement with these studies. The potential explanation could be that smoking contributes to a reduction in nitric oxide (NO) [36], and it has been speculated that reduced NO levels may protect smokers from some AMS related symptoms [35]. However, this phenomenon would probably only last for a short period and perhaps reduce long-term adaptation to high altitude [35].

Reports on the effect of gender on AMS have been mixed and inconclusive [28, 37], as we did not find any indication of gender differences with AMS. Some previous studies conducted among Himalayan trekkers [38], conference attendees [6] and mountaineers [8] have reported that age was inversely correlated with AMS. Our findings corroborate with this. One theory [39] about the relationship between age and susceptibility to AMS is that there are age-dependent differences in intracranial and intraspinal cerebrospinal fluid capacity [40]. Old people with a larger ratio of cranial cerebrospinal fluid to brain volume results in them being better able to compensate for brain swelling by a displacement of cerebrospinal fluid, and are less likely to suffer from AMS than young people with a lower ratio [40].

Since there were only a few other accommodations or guest houses for tourists that were not on the tourist bureau’s list, we have recruited participants from randomly selected hotels that represent the absolute majority of the places where tourists can stay in Lhasa. Most of the tourists who were invited to participate in the present study were willing to give the information that was asked for. We do not see strong reasons as to why AMS prevalence should be over- or underestimated in the study population. A more general problem with all such surveys is the potential selection mechanisms for the visiting populations, which could influence prevalence of AMS and associations between exposure and AMS. For example, it seems reasonable that people’s decision not to go high could be affected by a previously bad experience with AMS. We believe that our finding of no negative effect of a former experience with AMS could have been caused by self-selection. Such selection processes could also affect other associations. It could be that people in old age, people who do not take prophylactic medicine, people with diseases, as well as smokers who decide to go high are a “healthy” part of that exposure group, and that such characteristics may change over time and between populations. Consistency in findings between different study populations with different characteristics would be of help. However, there are reasons for expecting differences between studies, so to draw general causal claims from these types of studies is therefore challenging. As a consequence, we believe that our findings reflect conditions and relations among tourists in Tibet recently, and if these findings corroborate well with results from similar studies carried out elsewhere, one could expand the interpretation of the findings.

## Conclusion

AMS and AMS-related symptoms in tourists travelling to Lhasa are common, and tourists often need to reduce their activities during the first days of their stay. Symptoms typically start within the first 12 h after arriving. Associations between risk factors and AMS could be affected by self-selection in these types of studies. Age was inversely related to AMS, while country of origin, gender and reports of suffering from a chronic disease did not seem to be predictors of AMS. Subjects planning to visit high-altitude areas should be prepared for experiencing AMS-related problems, and consider preventive measures such as pre-exposure or gradual ascent to high altitudes.

## Abbreviations

95 % CI: 95 % confidential interval
AMS: acute mountain sickness
BMI: body mass index
HACE: high-altitude cerebral edema
HAPE: high-altitude pulmonary edema
LLSS: Lake Louise Scoring System
PR: prevalence ratio
TAR: Tibet Autonomous Region
